# A NOTCH-sensitive uPAR-regulated oncolytic adenovirus effectively suppresses pancreatic tumor growth and triggers synergistic anticancer effects with gemcitabine and nab-paclitaxel

**DOI:** 10.18632/oncotarget.15169

**Published:** 2017-02-07

**Authors:** Ana Mato-Berciano, Giulia Raimondi, Maria Victoria Maliandi, Ramon Alemany, Lluis Montoliu, Cristina Fillat

**Affiliations:** ^1^ Institut d'Investigacions Biomèdiques August Pi i Sunyer (IDIBAPS), Barcelona, Spain; ^2^ Centro de Investigación Biomédica en Red de Enfermedades Raras (CIBERER), Barcelona, Spain; ^3^ Institut Català d'Oncologia-IDIBELL. L'Hospitalet de Llobregat, Barcelona, Spain; ^4^ Centro Nacional de Biotecnologia (CNB-CSIC), Madrid, Spain

**Keywords:** pancreatic cancer, oncolytic adenovirus, cancer stem cells, gemcitabine, nab-paclitaxel

## Abstract

Notch signaling pathway is an embryonic program that becomes reactivated in pancreatic cancer and contributes to cancer stem cell (CSC) maintenance. We explored the concept of oncolytic adenoviral activity in response to Notch activation signaling, in the context of a chimeric promoter with uPAR regulatory sequences, as a strategy to drive its activity in neoplastic and CSC. We explored the advantages of a chemo-virotherapy approach based on synergistic combinations. Regulatory sequences recognized by the transcriptional factor CSL upstream a minimal uPAR promoter were engineered in adenoviral vectors and in the oncolytic adenovirus AdNuPARmE1A. Viral response to Notch signaling, and viral potency in cell lines and pancreatic cancer stem cells (PCSC) was tested. Preclinical toxicity and antitumor efficacy in xenografts and Patient-derived xenografts (PDX) mouse models was evaluated, as unimodal or in combination with gemcitabine+nab-paclitaxel. Mechanistic studies were conducted to explore the synergism of combined therapies.

We demonstrate that CSL-binding site optimized-engineered sequences respond to Notch activation in AdNuPARmLuc and AdNuPARmE1A. AdNuPARmE1A showed strong lytic effects in pancreatic cancer cell lines and PCSC. AdNuPARmE1A displayed attenuated activity in normal tissues, but robust antitumor effects in xenograft and PDX models, leading to a reduced capacity of treated tumors to form tumorspheres. Chemo-virotherapy treatment enlarged therapeutic response in both tumor models. Synergistic effects of the combination resulted from viral sensitization of apoptotic cell death triggered by chemotherapy.

In summary we present a novel effective oncolytic adenovirus, AdNuPARmE1A that reduces PCSC and presents synergistic effects with gemcitabine and nab-paclitaxel, supporting further clinical development.

## INTRODUCTION

Reactivation of embryonic programs is a common characteristic of human malignancies. In pancreatic cancer reactivation of Hedgehog, Wnt and Notch signaling pathways is well defined [1–4]. Notch signaling acts as a mediator of growth regulatory pathway and as a regulator of the balance between self-renewal and differentiation in the developing pancreas [5]. In pancreatic cancer Notch activity has been shown to synergize with K-ras, promoting PanIN initiation and progression, and to contribute to the maintenance of the pancreatic CSC population [6, 7]. The Notch pathway initiates when a cell expressing the appropriate ligand (jagged or delta) interacts with another cell expressing a Notch receptor (NOTCH1-4). Upon ligand binding the transmembrane receptor is cleaved and releases the intracellular domain of Notch (NICD) which translocates into the nucleus where it interacts with the DNA binding factor RBP-J, also known as CSL, recruits co-activators and turns on transcription of target genes [8]. Inhibitors of the Notch pathway have been tested in preclinical models and shown antitumor effects. Currently several inhibitors are under clinical trials in combination with chemotherapy for advanced-stage Pancreatic Ductal Adenocarcinoma (PDAC) [9].

The devastating nature of PDAC, is the fourth most common cause of death from cancer, encourages the need towards the development of novel therapies and the identification of synergisms between treatments.

Oncolytic adenoviruses, designed to replicate, spread and lyse tumor cells are under evaluation in clinical trials for PDAC treatment. First results from phase I/II clinical trials with the E1B-deleted adenovirus ONYX-15 in combination with gemcitabine showed the safety of the treatment but clinical efficacy was modest [10]. Improvement on viral activity in the tumors is approached by means of a plethora of different strategies. Our group has previously developed the oncolytic adenovirus AduPARE1A, where the transcriptional regulation of the E1A gene, which drives viral replication, is under the control of the urokinase-type plasminogen activator receptor (uPAR) promoter. We have shown anticancer activity in several pancreatic cancer models as a unimodal treatment and a synergistic antitumor effect when combined with gemcitabine through an NF-kB mediated mechanism of uPAR promoter activation [11–13].

One strategy to enhance the oncoselectivity of an adenovirus is to take advantage of the transcriptional reprogramming that takes place in tumor cells and that allows the reactivation of embryonic developmental pathways. Based on the sustained activation of the Notch signaling pathway in pancreatic cancer and their key role in tumorigenesis and pancreatic cancer stem cell maintenance, we hypothesized that an adenovirus engineered with a chimeric sequence comprising Notch-responsive elements combined with the uPAR promoter might improve the transcription of E1A in neoplastic and cancer stem cells, thus enhancing viral tumor activity and oncoselectivity. Here we demonstrate that a chimeric sequence with multiple CSL binding sites upstream of a minimal uPAR promoter is recognized by the CSL-NICD complex and shows sensitivity to Notch signaling activation, leading to increased transcription and strong activity in neoplastic and cancer stem cells. The oncolytic AdNuPARmE1A displayed potent replication in tumor cells, similar to Adwt, and showed strong antitumor activity and oncoselectivity. Synergistic effects were observed with gemcitabine and nab-paclitaxel *in vitro*. *In vivo*, we show for the first time that the combination treatment of AdNuPARmE1A and the gold standard chemotherapy treatment for PDAC led to tumor regression in xenograft and PDX models and decreased the pancreatic cancer stem cell content in treated tumors.

## RESULTS

### Adenoviruses engineered with CSL-binding sites respond to Notch pathway activation

**Figure 1:**
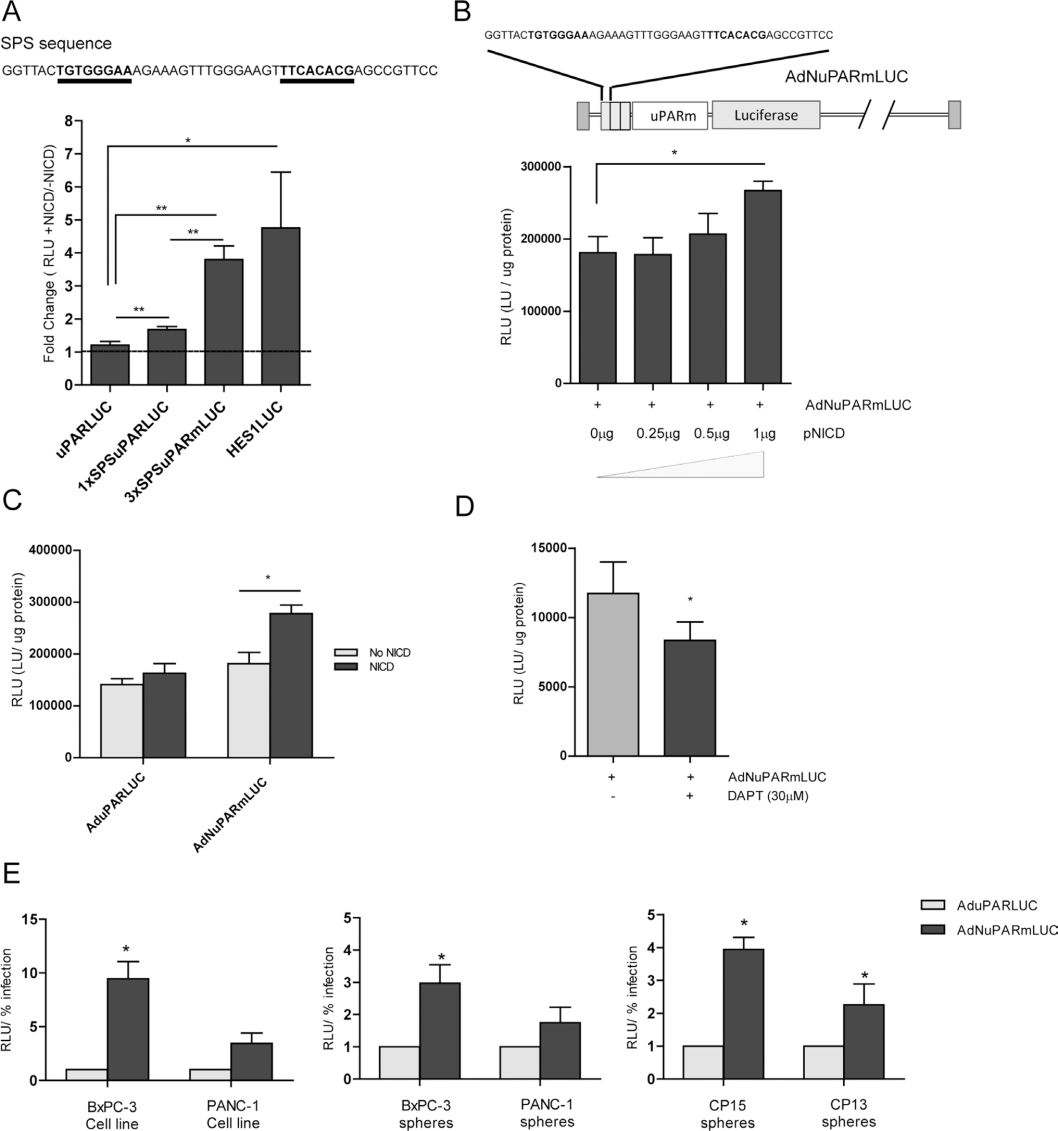
Notch-dependent transcriptional activity from the chimeric promoter controlled by the uPAR regulatory sequences and Notch responsive elements (**A**) Reporter plasmids that express the luciferase gene under the control of uPAR, 1xSPSuPAR or 3xSPSuPARm promoters were tested for luciferase activity in NIH/3T3 cells under the presence, or not, of NICD. Hes1LUC plasmid was used as positive control of Notch response. (**B**) Reporter adenovirus was designed using 3xSPSuPARm promoter (upper panel). MIAPaCa-2 cells were transduced with 10 MOI of Ad3xSPSuPARmLuc and then transfected with increasing amounts of pNICD encoding plasmid. Luciferase activity was analyzed 48 h after transfection. (**C**) Luciferase activity of MIAPaCa-2 cells transduced with AduPARLUC or AdNuPARmLUC (10 MOI) and transfected with 1 μg pNICD. (**D**) Luciferase activity in BxPC-3 cells transduced with AdNuPARmLUC (5 MOI) and treated with 30 μM of DAPT. Statistical differences were analyzed by Student's *t-test* (**E**) AduPARLUC and AdNuPARmLUC activity in several pancreatic cancer models: BxPC3 and PANC-1 cell lines (left panel), BxPC3 and PANC-1 spheres (middle panel) and CP15 and CP13 tumorspheres derived from PDX (right panel). All cells were transduced at 5 MOI and luciferase expression was analyzed at 48 h after transduction and normalized by the % of infection (% of GFP expressing-cells). Results are expressed as a mean +/− SEM of at least three independent experiments (*p < 0.05; **p < 0.01; ***p < 0.001).

Notch-responsive genes are characterized by a DNA-binding domain, recognizing the CSL transcription factor in the promoter region. The presence of dual “sequence-paired” CSL-binding sites (SPS) orientated head-to-head and separated by 16nt promotes the dimerization of the Notch transcriptional complex, leading to transcriptional activation of Notch target genes, such as Hes1[14]. We engineered a luciferase reporter plasmid with the uPAR promoter sequence containing one SPS sequence (1xSPS) upstream the promoter. We also constructed a reporter plasmid using a minimal uPARm promoter sequence (244pb), capable to promote the transcription activity [15], preceded by three SPS sequences (3xSPS). Transfection of the different plasmids, in combination with a plasmid expressing the Notch intracellular domain (pNICD), showed increased luciferase activity with the 3xSPS construct, similar to the levels achieved with the positive control HES1Luc plasmid, a well-defined Notch-responsive promoter (Figure 1A). On the basis of these data we generated a reporter adenovirus expressing the luciferase gene under the control of 3xSPSuPARm chimeric promoter and designated as AdNuPARmLuc. Cell transfection with pNICD showed a notch activation dependent activity of AdNuPARmLuc but not of the control virus AduPARLuc (Figure 1B, 1C). Furthermore, in the presence of the γ-secretase inhibitor DAPT AdNuPARmLuc showed reduced activity (Figure 1D). These data indicates that the 3xSPS sequences inserted in the adenoviral genome confer Notch-dependent activation of the chimeric promoter. Next, we analyzed the activity of AdNuPARmLuc with respect to AduPARLuc in pancreatic cancer cells and tumorspheres from cells and PDX tumors, expressing NOTCH receptors and the surrogate marker of Notch signaling pathway HES1 (Supplementary Figure 1). Increased luciferase activity was observed in all cells transduced with AdNuPARmLuc suggesting that Notch-responsive elements contributed to enhance promoter activity in pancreatic cancer (Figure 1E).

Next we generated an oncolytic adenovirus in which the E1A gene was under the control of the 3xSPSuPARm sequences. First we tested for an optimal construct that incorporates insulator sequences with enhancer-blocking activity and minimal size [16]. A 250 bp element of the core sequence of the chicken b-globin 5′ cHS4 locus (CORE) and the 214 bp short interspersed nuclear element B2 from the growth hormone boundary region (SINEB2) were inserted upstream the uPAR promoter controlling E1A, and the corresponding oncolytic viruses were generated [17, 18]. As an indirect measure of their insulation capacity, we determined the cytotoxic response of the different viruses to gemcitabine induced-activation and compared to the previously generated AduPARE1A bearing the insulator from the myotonic dystrophy locus (DM) (Supplementary Figure 2A). All the insulated viruses showed significant enhanced cytotoxicity triggered by gemcitabine (Supplementary Figure 2B). We have recently proposed that the increased cytotoxicity of AduPARE1A+gemcitabine combination is the result of NF-kB gemcitabine-mediated induction acting on the uPAR promoter [12]. In this line, the similar response of the three insulated viruses to gemcitabine treatment suggest that none of the insulator elements neither viral sequences were interfering on the uPAR promoter regulation.

**Figure 2:**
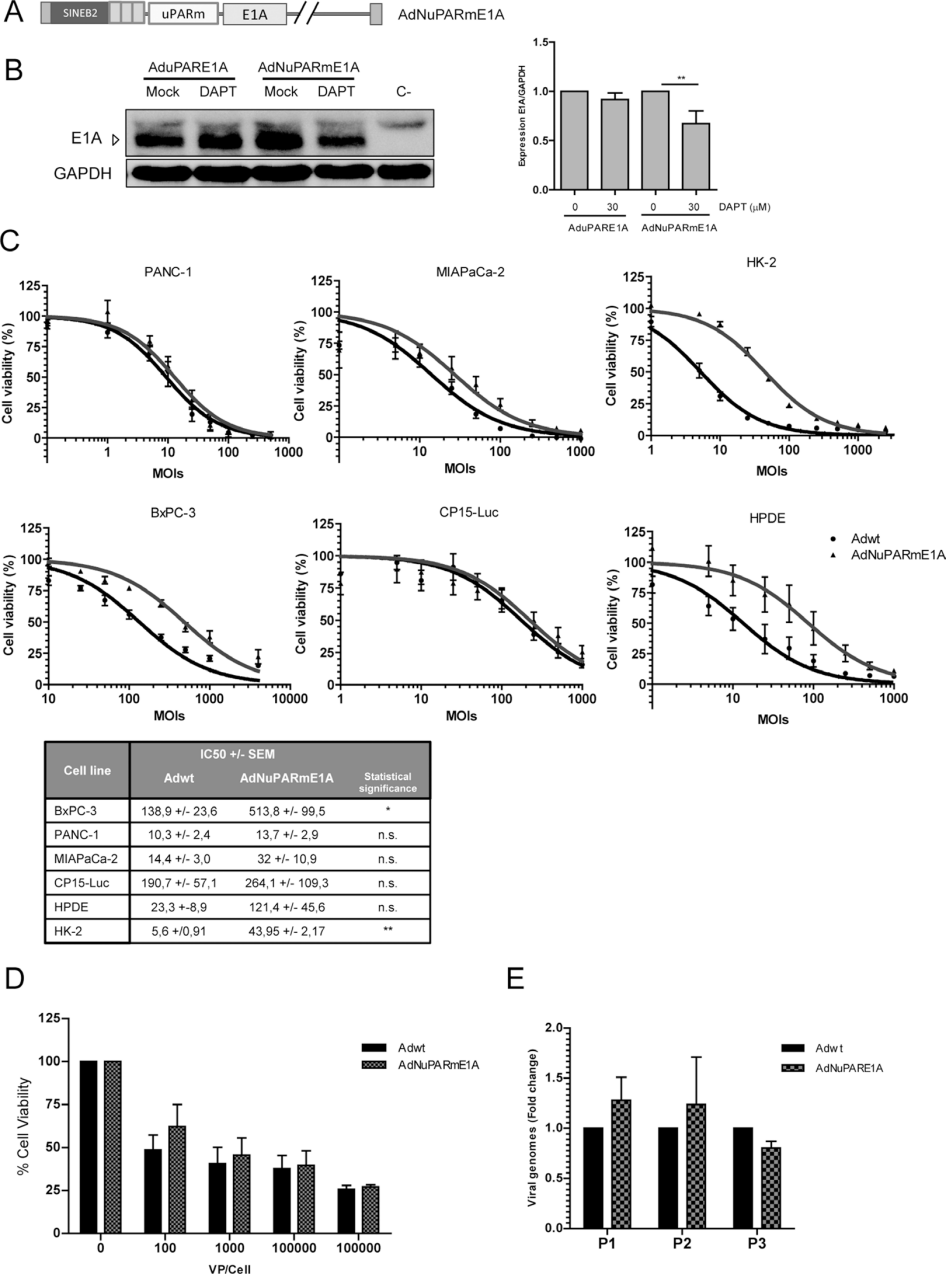
AdNuPARmE1A oncolytic adenovirus is regulated by the Notch pathway and displays a high oncolytic activity in pancreatic cancer models *in vitro* (**A**) Schematic representation of the oncolytic adenovirus AdNuPARmE1A. Expression of the E1A adenoviral gene is controlled by the 3xSPSuPARm promoter. SINEB2 insulator sequence was cloned upstream the promoter sequence. (**B**) Western blot of E1A gene expression in BxPC-3 cells infected with AduPARE1A or AdNuPARmE1A (50 MOIs) and treated, or not, with 30 μM of DAPT for 48 h. Bar graph shows quantification of E1A expression normalized to GAPDH levels (right panel). Results are expressed as a mean +/− SEM of *n* = 7 independent experiments (*p < 0.05; **p < 0.01). (**C**) *In vitro* oncolytic activity of AdNuPAREmE1A compared to Adwt in PANC-1, BxPC-3, MIAPaca-2, CP15-Luc HPDE and HK-2 cell lines. Cells were seeded in triplicate and treated with a dose range of adenoviruses (vp/cell). Cell viability was measured 72 h post-infection by MTT assay and normalized to mock treated cultures. IC50 mean values +/− SEM of at least four independent experiments are represented at the table (*p < 0.05; **p < 0.01). (**D**) *In vitro* oncolytic activity of AdNuPAREmE1A compared to Adwt in CP15 tumorspheres. Cell viability was measured 72 h post-infection by WST-1 assay and normalized to mock treated cultures (*n* = 3 independent experiments). (**E**) Quantification of viral production in CP15 tumorspheres infected with AdNuPARmE1A or Adwt upon several passages by qPCR. Results are expressed as the mean of relative viral genomes +/− SEM of three independent experiments.

Because there is a limitation for the size of the adenoviral genomes that can be packaged into viral particles, the smallest insulator that corresponded to the SINEB2 sequence was chosen to generate AdNuPARmE1A (Figure 2A). The new virus also showed increased cytotoxicity in the presence of gemcitabine that was synergistic, as previously reported by AduPARE1A (Supplementary Figure 3). Importantly, AdNuPARmE1A was sensitive to Notch signaling since in the presence of the DAPT γ-secretase inhibitor, E1A expression was significantly reduced. This effect was not observed in the AduPARE1A virus that lacks the Notch-responsive elements (Figure 2B). These data were indicative that the presence of the novel insulator SINEB2 resulted in good promoter fidelity.

### AdNuPARmE1A displays strong cytotoxic activity in neoplastic and cancer stem cells

To evaluate virus replication potency and tumor selectivity, a panel of pancreatic cancer cell lines, the non-tumoral pancreas-derived HPDE and the non-tumoral epithelial kidney HK-2 cells were infected with AdNuPARmE1A or the control virus Adwt at different viral dilutions, and cell viability was measured. AdNuPARmE1A induced similar cytotoxicity to Adwt with IC50 values ranging from 3.7 to 1.3-fold compared to the parental virus. In contrast, in non-tumoral HPDE and HK-2 cells AdNuPARmE1A displayed significantly reduced cytotoxicity than Adwt (Figure 2C, Supplementary Figure 4). Similar activity of the two viruses was also observed in tumorspheres from the patient-derived tumor CP15 (Figure 2D). Viral release in tumorspheres was also similar between the two viruses after consecutive rounds of infection (Figure 2E).

Therefore, AdNuPARmE1A showed similar potency to Adwt *in vitro* in cancer cell lines and tumorspheres but enhanced selectivity.

Furthermore, the degree of selectivity of the AdNuPARmE1A was superior to that of AduPARE1A since both viruses displayed similar cytotoxicity in cancer cells but AdNuPARmE1A showed reduced activity in non-tumoral HPDE and HK-2 cells (Supplementary Figures 4, 5). These data suggest that despite the selective activity of the two insulated viruses was preserved, the SINEB2 insulator may confer enhanced selectivity.

### AdNuPARmE1A shows a low toxic profile upon systemic administration in inmunocompetent mice and triggers strong antitumor responses in xenograft and PDX pancreatic cancer models

**Figure 3:**
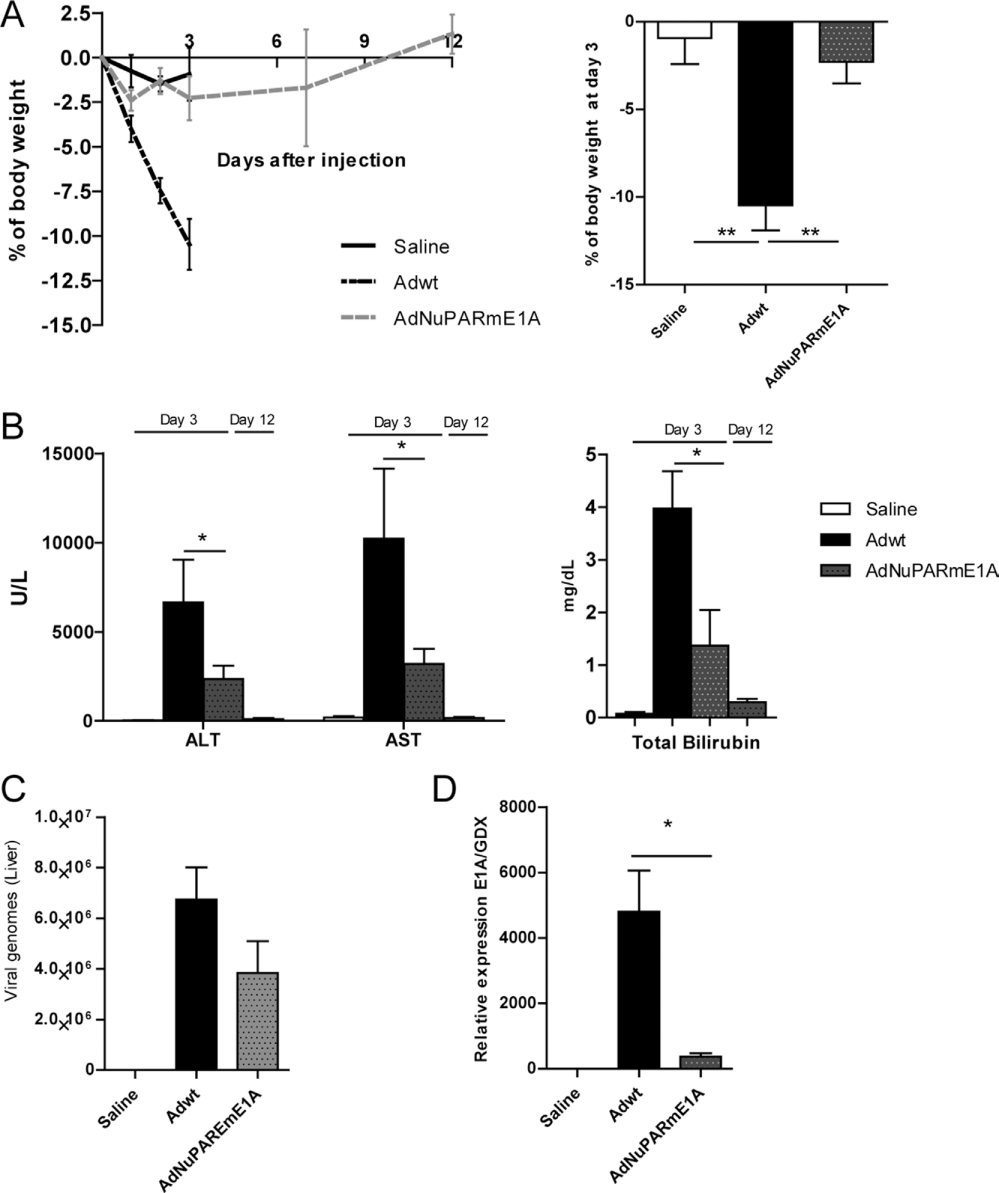
Toxicity profile of AdNuPARmE1A after systemic administration in immunocompetent mice (**A**) Percentage of body weight variation in immunocompetent mice after intravenous administration of Adwt or AdNuPARmE1A (5·10^10^ vp/mouse), or saline in the control group. Bar graph shows the percentage of body weight variation at day 3 post-administration (right panel). (**B**) Assesment of hepatotoxicity by the determination of AST, ALT and total bilirubin in the serum of treated mice. (**C**) Viral genomes production in livers of treated mice. (**D**) qPCR of E1A mRNA expression in livers of treated mice, relative to GDX expression. Results are expressed as the mean +/− SEM of *n ≥* 8 animals/group, or *n* = 5 in the saline control group. (*p < 0.05; **p < 0.01).

To investigate the oncolytic activity and oncoselectivity of AdNuPARmE1A *in vivo*, we first studied the impact on liver-damage associated toxicity induced by systemic delivery of adenovirus. Mice received intravenously 5 × 10^10^ vp of AdNuPARmE1A or Adwt and we measured the body weight loss and the release of transaminases and total bilirubin to the serum, as indicators of liver toxicity. Adwt, triggered a 25% mortality and a major loss of weight by day 3. In contrast, a slight non-significant decrease was observed by day 3 in AdNuPARmE1A injected mice that completely recovered by day 12 (Figure 3A). A robust increase in AST, ALT and bilirubin was detected in Adwt injected mice at day 3, whereas significantly less induction was detected in AdNuPARmE1A animals and normalized by day 12, showing similar levels than saline control group (Figure 3B).

These results are consistent with the slightly reduced number of viral genomes (Figure 3C) and the diminished expression of E1A in the mouse liver (Figure 3D), as a result of the low activity of the insulated chimeric tumor specific promoter NuPARm in non-tumoral cells.

Comparative studies of liver toxicity after AduPARE1A or AdNuPARmE1A intravenous administration showed significantly low levels of transaminases by both viruses compared to Adwt mice, with AdNuPARmE1A displaying a safer profile (Supplementary Figure 6). These results are in line with the *in vitro* selectivity studies.

**Figure 4:**
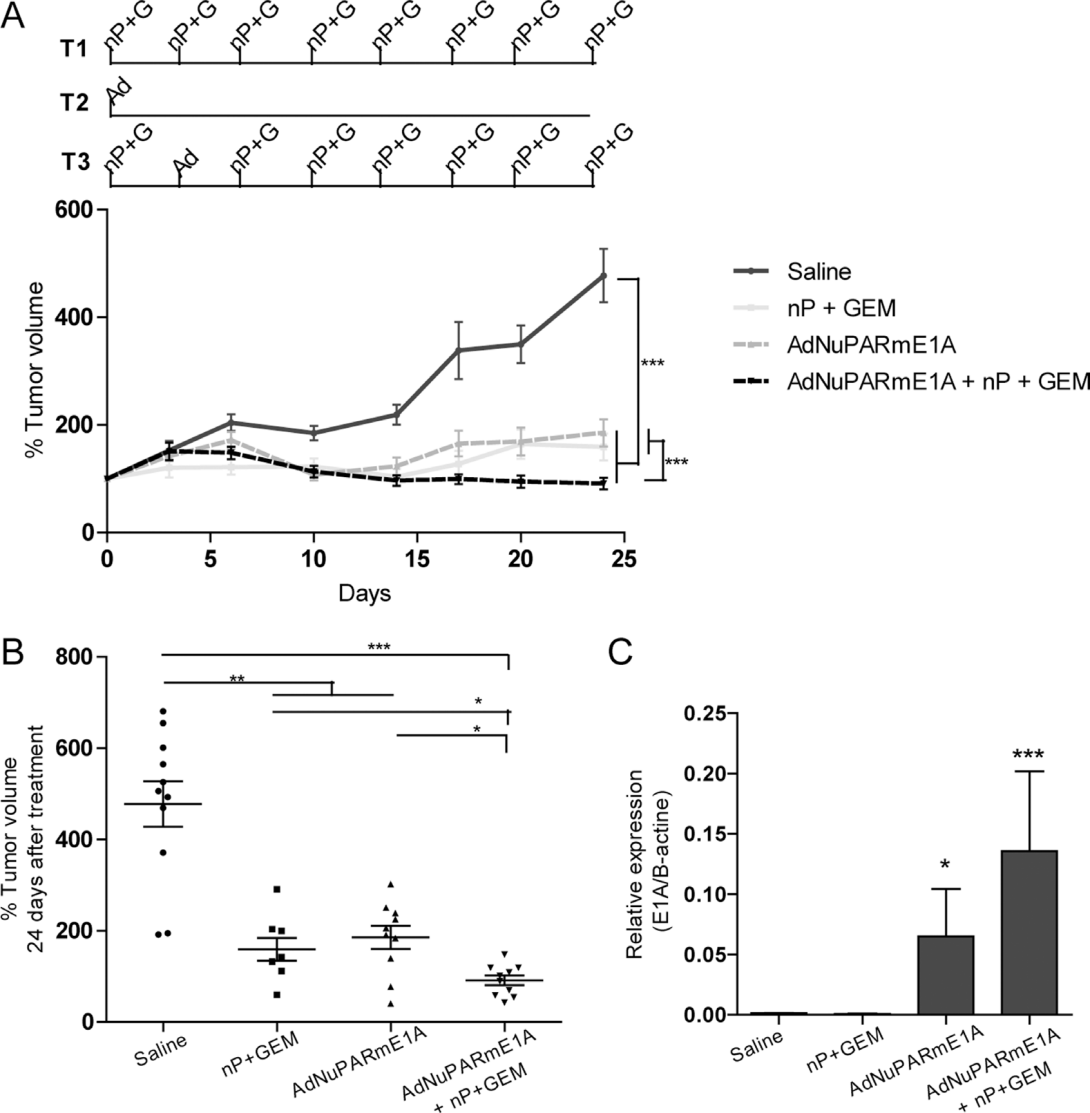
*In vivo* antitumoral activity of AdNuPARmE1A as a single agent or in combination with gemcitabine and nab-paclitaxel in MIAPaCa-2 tumors (**A**) Mice bearing subcutaneous MIAPaCa-2 tumors were treated with gemcitabine (100 mg/kg) and nab-paclitaxel (30mg/kg) twice a week (T1), with a single dose of AdNuPARmE1A (5·10^10^vp) (T2), with the combined treatment (T3) or with saline in the control group, (upper panel). Follow-up of tumor growth was monitored every other day for 25 days (lower panel). (**B**) Percentage of tumor volume (mm^3^) of saline and treated tumors at the end of the experiment. (**C**) qPCR of E1A mRNA expression in MIAPaCa-2 treated tumors normalized to β-actin levels. Results are expressed as the mean +/− SEM of at least *n* = 8 tumors/group (*p < 0.05; **p < 0.01; ***p < 0.001).

**Figure 5:**
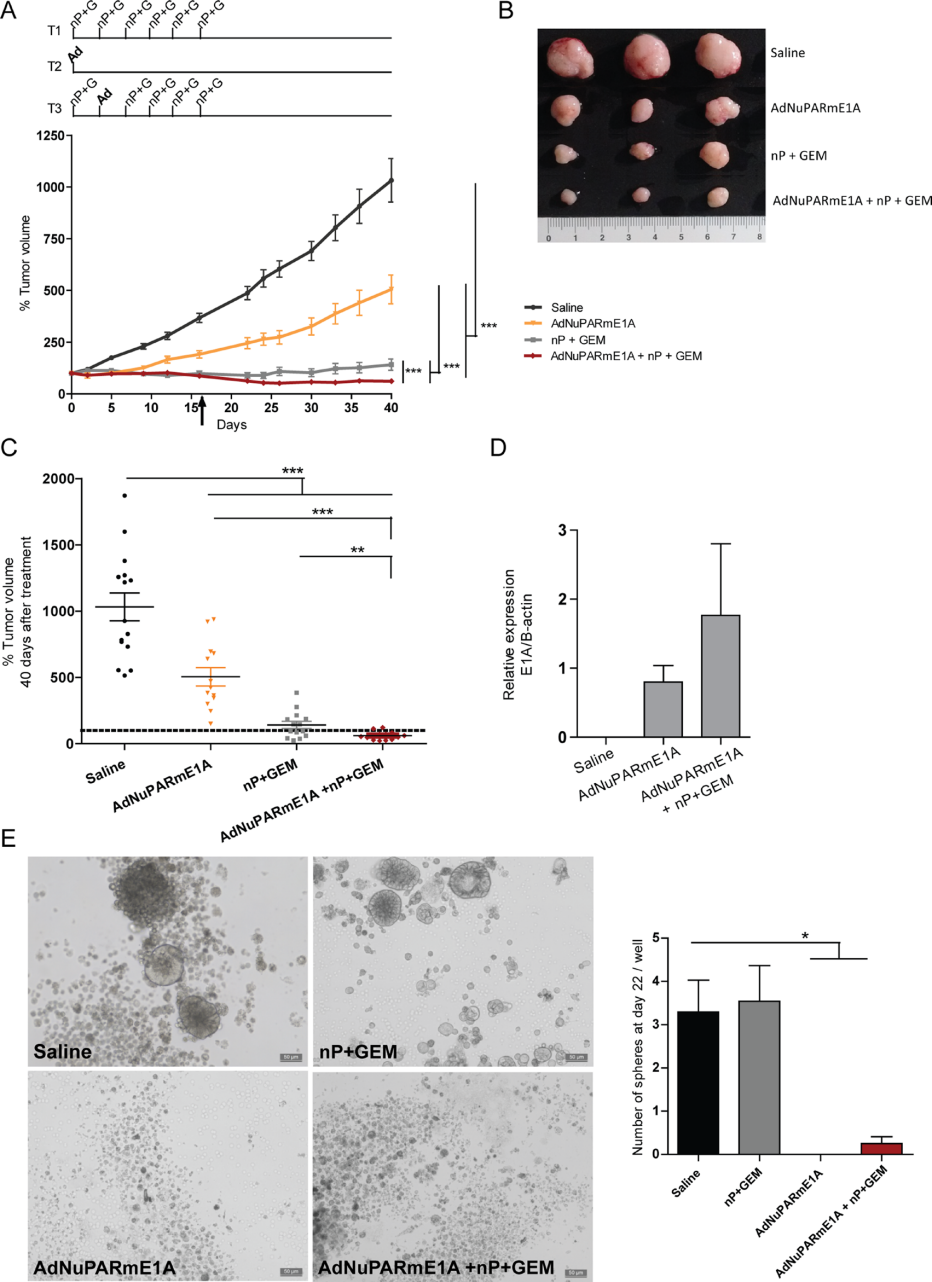
*In vivo* antitumoral activity of AdNuPARmE1A as a single agent or in combination with gemcitabine and nab-paclitaxel in CP15 PDX tumors (**A**) Mice bearing subcutaneous tumor fragments of CP15 PDX tumors were treated with gemcitabine plus nab-paclitaxel (T1), with a single dose of AdNuPARmE1A (5·10^10^vp) (T2), with the combined treatment (T3) or with saline in the control group, arrow indicates the day when the chemotherapy was stopped (upper panel). Follow-up of CP15 PDX tumor volume (mm^3^) represented as percentage of growth (lower panel), (*n ≥* 12 tumors/treatment group). (**B**) Image of three representative CP15 tumors for each treatment group. (**C**) Percentage of tumor volume (mm^3^) of CP15 treated tumors at the end of the experiment. (**D**) qPCR of E1A mRNA expression in CP15 treated tumors normalized to β-actin levels (**E**) Tumorspheres grown as anchorage-independent colonies from CP15 treated tumors, representative images (left panel), quantification of the number of tumorspheres (right panel). Results are expressed as the mean +/− SEM of *n* = 4 tumors/treatment group at 22 days after plating (*p < 0.05; **p < 0.01; ***p < 0.001).

AdNuPARmE1A systemic administration into mice bearing tumors from MIA PaCa-2 xenograft and CP15 and CP13 PDX models showed antitumoral effect. A good control of tumor growth was observed in the MIA Paca-2 model with stabilization of the tumor size throughout the study (Figure 4A, 4B). In the PDX models a reduction in tumor progression was achieved (Figure 5A–5C; Supplementary Figure 7A). Interestingly AdNuPARmE1A seem to also act on pancreatic cancer stem cells *in vivo*, since from viral-treated tumors it was observed a significative reduction in tumorospheres-forming capacity (Figure 5E, Supplementary Figure 7B). We confirmed that tumorspheres displayed stem-cell features since they were highly enriched in stem cell factors, such are the pluripotency-associated genes OCT 4, SOX2 and NANOG (Supplementary Figure 7C).

### Combination of AdNuPARmE1A with gemcitabine and nab-paclitaxel leads to synergistic effects and improves therapeutic outcome in xenografts and PDX models

We have recently shown that gemcitabine induces synergistic effects with AduPARE1A and this translates into improved antitumor efficacy of the combined treatment [12]. In the clinical setting, nowadays gemcitabine is administered in combination with nab-paclitaxel for metastatic PDAC. In this regard we tested whether the triple combination AdNuPARmE1A plus gemcitabine and nab-paclitaxel could provide any benefit over the chemotherapeutic regimen or the virus alone. MIA PaCa-2 xenografts were treated with virus i.v., the chemotherapeutic regimen of gemcitabine and nab-paclitaxel (GEM+nP) twice a week for 4 weeks or by the triple combination regimen in which the second dose of GEM+nP was substituted by i.v. AdNuPARmE1A (Figure 4A). Virus alone and the chemotherapeutic regimen had a similar efficacy to control tumor growth. The antitumor effect was maximal in the triple combination treatment, with regression of some tumors (Figure 4A, 4B). At the end of the experiment we measured expression of E1A in the tumors that received viral treatment and we observed increased E1A in the combined regimen, suggesting potentiation of the chemotherapy to the viral activity, and the potential of the therapeutic benefits to be extended if followed long-term (Figure 4C).

Similar experiments were also conducted in a more relevant model of pancreatic cancer, such as the CP15-PDX. Chemotherapy treatment was stopped by day 16 but tumor growth was follow up to day 40. Groups receiving chemotherapy either alone or in combination with AdNuPARmE1A showed a similar antitumor efficacy, during the treatment period. Although both groups displayed a good control in tumor growth until the end of the experiment, the group treated with the triple combination showed higher efficacy (Figure 5A). Macroscopic analyses evidenced a clear reduction in tumor size especially in the groups of the chemotherapy and triple combination treatments (Figure 5B). The largest reduction in tumor volume was observed in the virus plus chemotherapy regimen (Figure 5B, 5C). Analysis of E1A expression in the tumors, revealed that we could still detect adenoviral activity after 40 days treatment and a slightly increased in E1A expression was observed in the triple combination treatment (Figure 5D). We also assessed the effects of the treatment on the pancreatic cancer stem cell population by analyzing tumorsphere formation from the treated tumors. Tumorsphere forming capacity was significantly impaired in tumors treaded with AdNuPARmE1A either alone or in combination. However, chemotherapy treatment alone led to tumorsphere formation at similar numbers as the saline group (Figure 5E). These results demonstrate the abilitiy of the chimeric promoter NuPARm to induce the viral replication and the cell death in pancreatic neoplastic cells as well as in cells with stem cell features. Thus, the combined treatment AdNuPARmE1A + GEM+nP resulted in the largest antitumor effect and was able to target pancreatic cancer stem cells.

**Figure 6:**
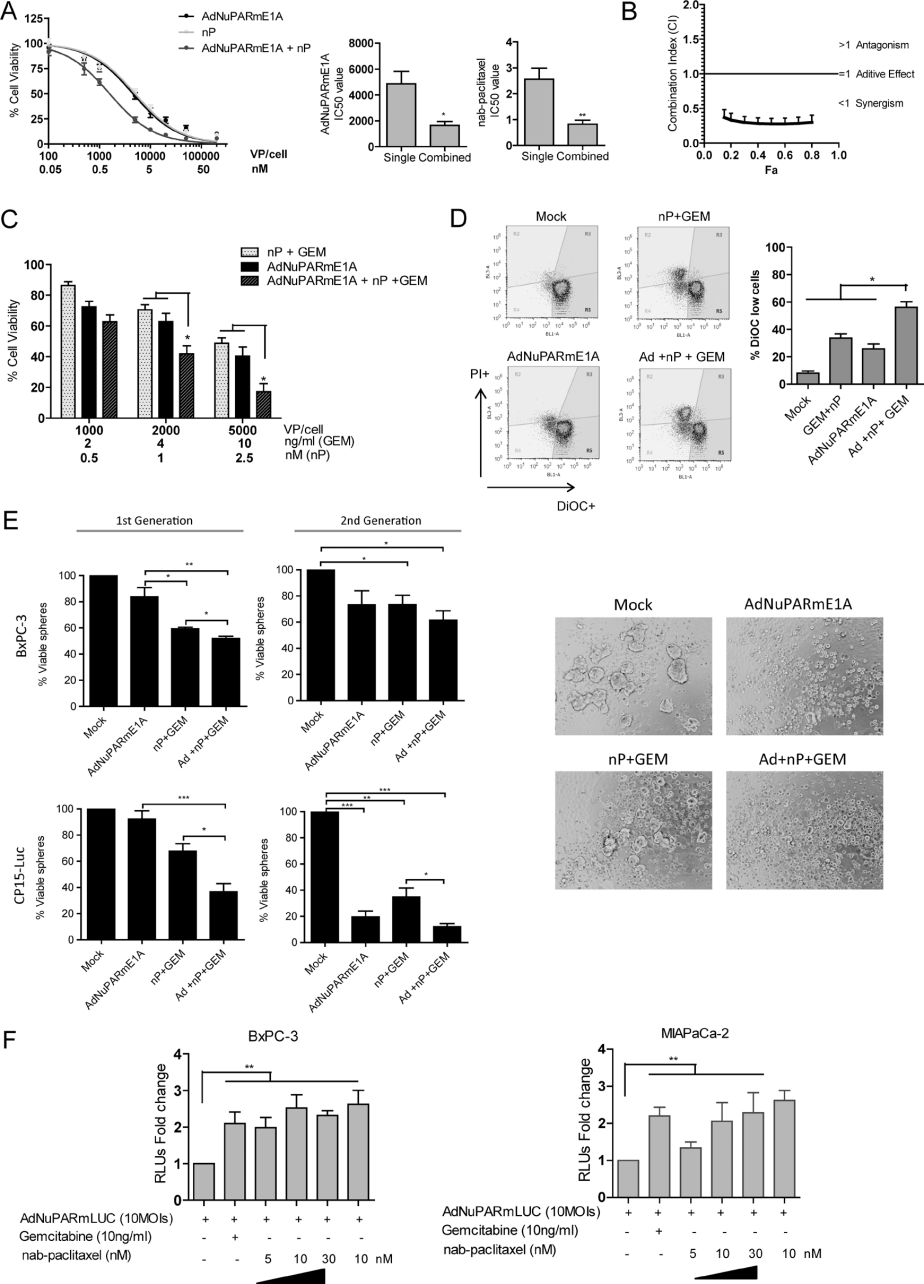
Cytotoxic and synergistic effects of the combination of AdNuPARmE1A with gemcitabine plus nab-paclitaxel in cellular models (**A**) Dose –response curves of nab-paclitaxel, AdNuPARmE1A or combined treatment in BxPC-3 cell line. Cells were seeded in triplicate and treated with a dose range of nab-paclitaxel (nM) and/or AdNuPARmE1A (vp/cell). Cell viability was measured 72 h later by MTT assay and is expressed as cell viability normalized to mock treated cultures. IC50 values for monotherapy or combination therapy for each treatment are represented in bar graphs. (**B**) Combination index values (CI) for the interaction of nab-paclitaxel and AdNuPARmE1A are calculated as a function of inhibitory fractions. Results are expressed as a mean +/− SEM of five independent experiments. (**C**) Cell viability in BxPC-3 cells treated with gemcitabine plus nab-paclitaxel, AdNuPARmE1A or combined treatment. Cells were seeded in triplicate and treated with several doses of nab-paclitaxel (nM) plus gemcitabine (ng/ml) and/or AdNuPARmE1A (vp/cell). Cell viabilitiy was measured 72 h later by MTT assay. Cell viability percentages relative to mock treated cells are represented in bar graphs as the mean +/− SEM of four independent experiments. Statistical differences were analyzed by Student's *t-test*. (**D**) Loss of mitochondrial transmembrane potential (ΨΔ_m)_ analysis in BxPC-3 cultured cells treated with AdNuPARmE1A (4000 vp/cell), with 50 ng/ml of gemcitabine plus 10 nM of nab-paclitaxel or with the combined treatment for 48 h, and staining with 15 nm 3,3*′*-diexyloxacarbocyanine iodide (DiOC_6_(3)). (**E**) Cell viability in BxPC-3 and CP15-Luc spheres treated with gemcitabine plus nab-paclitaxel, AdNuPARmE1A or combined treatment. Single cell suspensions of BXPC-3 and CP15-Luc cells were seeded at a density 2.0.10^4^ cells/mL with appropriate medium. Forty-eight hours later, BxPC-3 and CP15-Luc spheres were treated with nP 25 nM or 50 nM, gemcitabine 5 ng/mL or 2.5 ng/ml, AdNuPARmE1A 5000 vp/cell or 1000 vp/cell respectively alone or in combination and 72 h later, the percentage of viable spheres (1st generation) was determined by MTT assay. Then, 1st generation spheres were dissociated to single cell suspensions, re-plated at a density of 2.0.10^4^ cells/mL and 72 h later, CP15-Luc 2nd generation spheres images (10× magnification) were captured and the percentage of viable cells was determined by MTT assay. Statistical differences were analyzed by Student's *t-test*. (**F**) Luciferase activity in BxPC-3 and MIAPaCa-2 cell lines transduced with reporter adenovirus AdNuPARmLuc (10 MOIs) and treated with gemcitabine and/or nab-paclitaxel for 24 h. Results are expressed as the mean +/− SEM of at least four independent experiments. (*p < 0.05; **p < 0.01; ***p < 0.001).

We next investigated the potential mechanism of the improved antitumor effects of the triple combination treatment. Since we have previously shown that AduPARE1A and gemcitabine had a synergistic effect we explored whether the AdNuPARmE1A was also having any synergism with nab-paclitaxel. BxPC-3 cells were infected with increasing doses of viral particles and exposed to several concentrations of nab-paclitaxel either alone or in combination and cell viability was calculated 72 h later. A dose-response effect was observed for all treatments. Combination treatment significantly reduced the IC50 of each agent (Figure 6A). Pharmacological interaction between treatments assessed by the Combination Index (CI) analysis revealed a CI lower than 1 at all the fractions analyzed indicating synergism between treatments (Figure 6B). Analysis of the cytotoxicity produced by the virus alone, the GEM+nab-paclitaxel combination or the triple combination AdNuPARmE1A+ GEM+nab-paclitaxel on BxPC-3 cultures revealed a dose-response effect with the highest cytotoxicity in the combination treatment (Figure 6C). Increased apoptotic index was observed with chemotherapy and the triple combination therapy by measuring the changes in the mitochondrial transmembrane potential (ΔY), with the fluorescent loss of DIOC_6_(3) (Figure 6D). The enhanced response of the triple combination was also observed in spheres derived from BxPC-3 and CP15-Luc cells (Figure 6E, 1st Generation). Viability was highly compromised in second generation spheres, and the effect was more pronounced following AdNuPARmE1A+ GEM+nab-paclitaxel treatment. Almost complete prevention of CP15-Luc secondary sphere formation was observed in the AdNuPARmE1A and triple combination groups (Figure 6E, 2nd Generation).

Thus the increased efficacy of the triple combination treatment will rely on the synergistic effects that both gemcitabine and nab-paclitaxel display when combined with the AdNuPARmE1A. Interestingly, both chemotherapeutic agents had the ability to enhance the luciferase activity from AdNuPARmLuc in BxPC-3 and MIA PaCa-2 transduced cells, suggesting NuPARm promoter transcriptional activation by the chemotherapeutic agents (Figure 6F).

## DISCUSSION

The regulation of oncolysis according to the transcriptional reprogramming of embryonic activators is an interesting strategy for viral selectivity and to improve tumor activity. To our knowledge, no embryonic programs re-activated in pancreatic cancer have been tested to provide adenovirus with oncoselectivity besides the Wnt/b-catenin pathway that has been explored as an approach to target viral specificity to colorectal and pancreatic tumors with very strong antitumor responses [19, 20]. In the current work we considered the aberrant regulation of the Notch pathway in pancreatic tumors as an attractive target to develop a tumor-specific therapy based on oncolytic treatments. Here we have used for the first time a synthetic promoter engineered with sequences that respond to Notch signaling activation and a minimal uPAR promoter. We have demonstrated the ability of a Notch-dependent promoter to induce the viral activity. The newly generated virus demonstrated strong activity in pancreatic cancer cells, as well as in pancreatic cancer stem cells, in line with an overactivation of the Notch pathway in the PCSC compartment [6]. The oncolytic AdNuPARmE1A displayed robust responses both *in vitro* and *in vivo*. Interestingly, the activity of the virus in the PCSC compartment was also demonstrated *in vivo* with the impaired capacity of cells from treated tumors to grow in culture as tumorspheres. This is a remarkable phenomena since current treatment, based on cytotoxic chemotherapies, are known to show efficacy against the bulk of the tumors, but not in the resistant PCSC population, and lead, ultimately, to the failure of the treatment. In this line, we have observed that after the chemotherapeutic regimen of gemcitabine plus nab-paclitaxel, tumorspheres from treated tumors could be grown and expanded similarly to non-treated ones, suggesting the lack of efficacy of the therapy in this cell compartment. However, it has been described that chemotherapy when combined with Notch inhibitors resulted in synergistic effects [21], indicating that the combination of therapies acting on PCSC and in the bulk of the tumors hold great promise for cancer control.

Accordingly, AdNuPARmE1A and the chemotherapeutic regimen of gemcitabine+nab-paclitaxel in the MIA Paca-2 xenograft and in CP15 PDX tumors showed strong antitumor effects, at least in part as a consequence of viral lysis on the PCSC cells. Furthermore, we demonstrate that the remarkable anticancer effects observed *in vivo* may also account from an intracellular synergism between treatments.

The molecular basis of the synergism may rely on the ability of gemcitabine and paclitaxel to induce constitutive NF-kB activation [22, 23]. Activated NF-kB triggers a series of molecular reactions, with the upregulation of pro-survival genes, thereby evading apoptosis. Nevertheless, our data showed increased apoptosis in the combination treatment. Thus, the operating mechanism might be the result of both agents triggering the activation of NF-kB and the trapping of the NF-kB transcription factor to the uPAR sequences from the viral particles, that will act as a decoy system, hindering the activation of prosurvival genes, and sensitizing to gemcitabine+nab-paclitaxel chemotherapy-mediated apoptosis [12]. In this line, the transcriptional activation of the uPAR promoter by both chemotherapeutic agents was observed since increased luciferase activity from AdNuPARmLuc infected cultures was detected after gemcitabine or nab-paclitaxel treatment. However other scenarios could be envisioned. In fact, numerous reports have described cross-talk mechanisms between Notch and NF-kB pathways in diverse experimental models. In some context stimuli that activate NF-kB also lead to Notch activation. Gemcitabine, for instance, has been shown to induce NF-kB and to activate Notch signaling, although it is unknown whether the two pathways interact [23, 24]. In our context, the activation of the two pathways upon gemcitabine could in fact facilitate uPAR promoter transcription and in turn viral lytic activity. Other factors may also contribute to explain the synergism of gemcitabine and Nab-paclitaxel with AdNuPARmE1A. In this line, several authors have previously demonstrated that the increased in E1A expression by DNA damaging agents results in E1A-induced apoptosis [25–27]. Interactions of early viral genes with cellular factors in the presence of gemcitabine has recently been shown to abrogate Chk-1-mediated checkpoint contributing to enhanced cell killing [28]. Alterations in the cell cycle control, with the induction of mitotic slippage have also been associated to paclitaxel sensitization to oncolytic adenovirus [29, 30]. Furthermore, *in vivo*, authors have shown remarkable disruption of tumor architecture following adenoviral treatment, associated with increased activation of metalloprotease MMP9, what would facilitate chemotherapy penetration in the tumor, thus contributing to superior effects in the combined treatment [31]. On the other hand, nab-paclitaxel administration has been also related with the disruption of the stroma in pancreatic cancer, so there is the possibility that combined treatment helps to improve adenoviral release within the tumor [32].

For clinical development, safety of the treatment is also a key issue. Our data shows that AdNuPARmE1A, provides a good safety profile, since the expression of E1A, the major trigger of liver-associated toxicity upon systemic administration of the virus, was highly reduced with respect the non oncoselective wild type virus. AdNuPARmE1A also displayed a safer profile than the oncoselective AduPARE1A, suggesting that the novel insulator SINEB2 preserves promoter fidelity. In consequence, at high viral doses, liver damage parameters of transaminases and bilirrubin were only moderately increased by AdNuPARmE1A, and in contrast to Adwt AdNuPARmE1A did not compromise mice survival neither body weight.

In conclusion, our preclinical studies have shown that AdNuPARmE1A presents antitumor activity in a Notch-responsive manner with activity in PCSC and the bulk of the tumor. The capacity of AdNuPARmE1A to synergize with the chemotherapeutic regimen of gemcitabine+nab-paclitaxel to enlarge therapeutic activity provides strong support for the clinical translation of AdNuPARmE1A +GEM+ nab-paclitaxel treatment to PDAC patients.

## MATERIALS AND METHODS

### Cell lines and patient samples

Human pancreatic tumor cell lines (BxPC-3, PANC-1, MIA PaCa-2), NIH3T3, HEK293, HK-2 and 293T were obtained from the American Type Culture Collection (ATCC, Rockville, MD, USA), and cultured following the ATCC recommendations. Luciferase-expressing cells CP15-Luc were established by transducing the parental cells CP15 cell line established from CP15 tumors [33, 34] with a recombinant retrovirus pLHCluc following standard procedures and selected in 0.2 mg/ml hygromycin. Human pancreatic ductal epithelial cells (HPDE) were kindly provided by Dr. FX Real (CNIO, Madrid, Spain). HPDE were cultured as reported [35]. Cells from the ATCC were immediately expanded and frozen. Every 2 months, cells were plated again from a frozen vial of the original batch. HPDE and CP-15Luc cells were treated similarly to ATTC cells. Interspecies contamination was tested by microscopic observation and by PCR routinely. Confirmation of cell morphology was carried out by microscopic observation. Cells were not authenticated by the authors with STR DNA profiling.

CP15 and CP13 tumors were derived from surgical samples of patients with pancreatic adenocarcinoma and perpetuated as xenografts in the pancreas of immunodeficient mice as previously described [33].

### Human pancreatic tumorsphere formation and culture conditions

Single-cell suspensions from CP15 and CP13 tumors or BxPC-3, CP15-Luc and PANC-1 cells were culture in DMEM-F12 Advanced supplemented with 0.4%FBS, 2 mM L-glutamine, 10 U/ml penicillin and 10 μg/mL streptomycin, 20 μg/mL gentamicin, B27 1x (Life Technologies, Inc), 5 μg/mL insulin (I0516, Sigma-Aldrich), 20 ng/ml of recombinant human epidermal growth factor (HuEGF, Life Technologies) and 20 ng/ml basic fibroblast growth factors (bFGF, BD Bioscience). Cells were plated onto 24-multiwell plates previously coated with 10mg/mL Poly-HEMA (Sigma- Aldrich). Spheres were enzymatically dissociated (Trypsin-EDTA) and subcultured for several passages.

### Plasmids and constructs

The uPARLuc construct [11] was cloned in pGEM-T vector system (uPARLUC). SPS sequence (CSL binding sites) 5′ GGTTACTGTGGGAAAGAAAGTTT GGGAAGTTTCACACGAGCCGTTCC 3′ were cloned upstream the uPARLUC. 1xSPSuPAR fragment was obtained by PCR amplification of the uPAR promoter using a tailed-primer containing SPS sequence. Complementary oligonucleotides containing two consecutive SPS sequences (2xSPS) (chemically synthesized and purified by Eurofins MWG Operon) were annealed and cloned upstream the 1xSPSuPARLUC, obtaining 3xSPSuPARLUC. uPAR promoter from the uPARLUCconstruct was reduced to 244bp to generate the minimal promoter uPARm. 3xSPSuPARmLUC was obtained by the replacement of uPAR for uPARm promoter.

SINEB2 and CORE sequences are the active insulator regions belonging to the Growth Hormone and β-globin of chicken insulators, respectively [17, 36]. Both sequences were obtained by PCR amplification from p1FeI and pELucCD plasmids, respectively. Plasmids were kindly provided by Dr. Montoliu. CORE and SINEB2 elements were subcloned in pGEMT7z plasmid upstream the uPAR promoter.

The expression vector of the Notch1 intracellular domain (pNICD) and the reporter plasmid HES1-LUC were kindly provided by Dra. Anna Bigas [37].

### Adenoviruses

Replication-defective adenovirus AduPARLUC express the firefly luciferase gene under the control of uPAR promoter and the GFP under the CMV promoter [11]. 3xSPSuPARmLUC construct was subcloned into a pAdTRack vectors, and reporter adenoviruses were generated by homologous recombination with the adenoviral genome. Replication-defective adenoviruses were propagated in HEK293 cells and purified by standard cesium chloride banding.

Human adenovirus serotype 5 (Adwt) and AduPARE1A have already been described [11].

CORE-uPARp, SINEB2-uPARp and the non-insulated uPAR promoter fragments were obtained by PCR amplification with tailed-primers containing homology arms to pShDMuPARE1A to generate the corresponding pShuttle adenoviral vectors pShCORE-uPARp, pShSINEB2-uPARp, pShuPARE1A vectors. Such vectors were recombined with Adwt genome following standard protocols to generate, pAdCOREuPARE1A, pAdSINEB2uPARE1A and pAduPARE1A non insulated.

The oncolytic adenovirus AdNuPARmE1A was generated by first cloning the 3xSPSuPARm promoter into a pShuttle vector and inserting the SINEB2 insulator upstream the promoter to generate pShSINE3x SPSuPARmE1A. Homologous recombination of pShSINE3xSPSuPARmE1A vector with the adenoviral genome, was performed following the standard protocol to generate pAdNuPARmE1A. Recombinant genomes were transfected in HEK293 cells and amplified in A549 cells and purified by standard cesium chloride banding. Adenoviral concentrations were determined by optical densitiy (vp/ml) and by plaque-forming units (pfu/ml). All viruses presented a similar ratio of vp/pfu.

### Reporter gene assays

#### With reporter plasmids

NIH/3T3 were co-transfected with the luciferase reporter plasmids uPARLUC, 1xSPSuPARLUC, 3xSPSuPARmLUC or HES1-LUC (150 ng/well) and with the pNICD expressing vector (50 ng/well), or with the empty vector, and with the β-gal reporter plasmid (45 ng/well). CalPhos mammalian transfection kit (Clontech, Takara Bio Company Inc.) was used for transfection, following the manufactures guidelines. 48 h after transfection cell lysates were analyzed for luciferase activity using Luciferase Assay System (Promega) and β-galactosidase activity was used as a control of transfection efficiency. Results are expressed as RLU (Relative light units).

### With reporter adenoviruses

MIAPaCa-2 cells were transduced with reporter adenoviruses AduPARLUC, Ad1xSPSuPARLUC or Ad3xSPSuPARmLUC at 10 MOI. 4 h after transduction the medium was removed and fresh medium was added. 24 h after transduction cells were transfected with pNICD expressing plasmid, or with the empty vector (as described above). 48 h after transfection cell lysates were analyzed for luciferase activity. Results are expressed as RLU (Relative light units: light units (LU) normalized to total protein levels)

BxPC-3 cells were preincubated with 30μM DAPT (Calbiochem, 565784) for 24 h, and then cells were transduced with Ad3xSPSuPARmLuc at 5 MOI. 4 h after transduction the medium was removed and fresh medium with DAPT was added. 48 h after transduction cell lysates were analyzed for luciferase activity.

BxPC-3 and PANC-1 cell lines and BxPC-3, PANC-1, CP15 or CP13 spheres were transduced with the reporter adenoviruses AduPARLUC or Ad3xSPSuPARmLUC at 5 MOI. 48 h after-transduction cells lysates were analyzed for luciferase expression and RLUs were normalized by the % of GFP-expressing cells.

### Cell viability assay

A total of 3·10^3^ cells (PANC-1, BxPC-3, MIAPaCa-2, CP15-Luc, HK-2 and HPDE) or 6·10^3^ cells (CP15 spheres) were seeded in triplicate and infected with Adwt, AduPARE1A or AdNuPARE1A at different doses. 4 h after infection medium was removed and fresh medium was added. Cell viability was measured at 72 h or 7 days after infection, using the MTT colorimetric assay (USB, Affymetrix, CA USA) or WST-1 assay (Roche Diagnostics, Basel, Switzerland) in the case of CP15 tumorspheres. Dose-response curves and IC50 values were obtained by a standard non-linear regression using GraphPad Prism software (CA, USA)

### Combination index analysis

BxPC-3 cells were seeded in triplicate (as described above) and treated with serial dilutions of nab-paclitaxel (nM) (Abraxane, Celgene), AdNuPARmE1A (vp/cell) or the combination of both treatments, maintaining a constant ratio of nab-paclitaxel:adenovirus of 1:2000. Cell viability was measured at 72 h after infection by MTT colorimetric assay, as described above. The induction of synergism, summation or antagonism between nab-paclitaxel and AdNuPARmE1A treatments was analyzed by combination index analysis, by the adapted method of Chou-Talay [38]. Dose-response curves and IC50 values were obtained by a standard non-linear regression using GraphPad Prism software (CA, USA) and combination index values were calculated using Calcusyn Software (Biosoft, Cambridge, UK). CI > 1 indicates antagonism, CI = 1 additivity and CI < 1 indicates synergism between treatments.

### Viral progeny production

CP15 spheres were tripsinized as single cells, seeded at a density of 6 × 10^3^ cell per well in poly-HEMA coated plates and infected with Adwt, or AdNuPAREmE1A at 1000 vp/cell. At 72 h post-infecction, 25% of the supernatant of infected cells was used to infect a new passage of tumorspheres. This was performed for 3 passages. Viral genome quantification was performed for all the passages. Viral DNA was obtained using the UltraClean BloodSpin DNA Isolation kit (Mo Bio Laboratories, Carlsbad, CA) according to the manufacturer´s instructions.

DNA from frozen liver tissue was obtained by incubating in a lysis buffer (100 mM NaCl, 10 mM TrisHCl pH8.0, 25 mM EDTA, 0,5% SDS) containing 0,1 mg/mL proteinase K overnight at 55°C, and then purified by phenol-clorophorm method.

Viral genomes were determined by qPCR using SYBR Green I Master mix (Roche Diagnostics, Basel, Switzerland) and the hexon primers (Supplementary Table 1) as described [39].

### Quantitative RT-PCR analysis of gene expression

Total RNA from tumors and cell cultures was isolated using RNeasy Mini kit (Qiagen). To avoid genomic contamination RNA samples were treated with DNAse (DNAfree, Ambion, Thermo Fisher Scientific) and reverse transcription reaction was performed to generate cDNA using the RETROscript kit (Ambion), according to manufacturer's instructions. Quantitative PCR reactions were performed using SYBR Green I Master mix (Roche Diagnostics, Basel, Switzerland) and 1 μl of cDNA and specific primers (Supplementary Table 1) with the ViiA7 System (Applied Biosystems).

### Western blot analysis

BxPC3 cells were seeded and infected with AduPARE1A or AdNuPARE1A (50 MOI). Four h after infection medium was removed and fresh medium with DAPT 30μM was added for 48 h. Total protein extracts were obtained with lysis buffer (50 mM Tris-HCl (pH6.8), 2% SDS and 10% Glicerol) containing Complete Mini Protease Inhibitor Cocktel (Roche Diagnostics, Basel, Switzerland). Cell lysates were boiled for 10 min at 98°C. Protein concentration was determined by BCA Protein Assay Kit (Thermo Fisher Scientific) and 80 μg of total protein was resolved in 8% SDS-PAGE and transferred to nitrocellulose membrane. Membranes were immunobloted with anti-adenovirus2/5 E1A polyclonal antibody (1:200, overnight at 4°C), or anti-GAPDH (1/3000, 1 h at room temperature), rinsed with TBS-Tween and then incubated with HPR-conjugated goat anti rabbit (1/2000, 1 h at room temperature). Antibody labeling was detected by ECL Western blotting detection reagent (Amersham, GE Healthcare, UK).

### Flow cytometry analysis

BxPC-3 cell cultures were treated with 4000 vp/cell of AdNuPARmE1A, with 50 ng/ml of gemcitabine plus 10 nM of nab-paclitaxel or with the combined treatment for 48 h. Loss of mitochondrial transmembrane potential (ΔΨ_m)_ was evaluated by staining cultured cells with 15 nm 3,3′-diexyloxacarbocyanine iodide (DiOC_6_(3)) (Molecular Probes, Eugene, OR, USA) and propidium iodide (PI) (Bender Medsystems). Flow citometry analysis was performed using the Attune Acoustic Focusing Cytometer and Software (Applied Biosystems). GFP-positive cells were quantified by flow cytometry BD FACSCanto II and FACSDiva software (Becton Dickinson, USA).

### Mouse xenografts and *in vivo* treatment

Male athymic BALB/c nude mice (8 weeks old, Harlan Iberica) were used to generate xenograft models. Subcutaneous tumors were generated by the injection of 2 × 10^6^ MIAPaCa-2 cells embedded in a ratio 1:1 with Matrigel (BD, Bioscience, USA) into each flank of nude mice, or by the implantation of 2 mm^3^ CP15 or CP13-patient tumor fragments. Tumors were measured and volumes were calculated according to the formula V (mm^3^) = 0.4 × (larger diameter · smaller diameter^2^). When tumors reached a tumor volume of approximately 100 mm^3^, animals were divided into four groups of treatment: Saline (T1), nab-paclitaxel plus gemcitabine (Hospira UK Limited) (T2), AdNuPARmE1A (T3), and the combined group nab-paclitaxel plus gemcitabine plus AdNuPARmE1A (T4). AdNuPARmE1A was i.v administrated as a single dose of 5 × 10^10^ vp/animal, and chemotherapeutic drugs were administrated twice a week for 3 weeks (CP15 PDX xenografts) or until the end of the experiment (MIAPaCa-2 model). Nab-paclitaxel was administrated i.v at 30mg/kg; Gemcitabine was i.p injected at 100 mg/kg 2 hours after nab-paclitaxel administration.

Animal procedures met the guidelines of European Community Directive 86/609/EEC and were approved by the Local Ethical Committee.

### Preparation of single-cell suspensions from human PDAC tumors

Isolation of single-cell suspension from CP15 and CP13 tumors has been previously described [13]. Briefly, tumor fragments were minced completely and incubated with 200 U/ml of collagenase IV (Sigma). After the elimination of death cells, cell suspension were filtered through a 70-μm and a 40-μm filter and collected in DMEM-F12 0.4% FBS.

### *In vivo* toxicity assay

C57Bl/6/129 mice were treated i.v. with a single dose of Adwt or AdNuPARmE1A (5 × 10^10^ vp/animal in a final volume of 100 μl) or saline in the control group and Adwt or AdNuPARmE1A or AduPARE1A (2 × 10^10^ vp/animal). Body weight was monitored for 3 days. Three days after viral administration blood samples were collected by intracardiac puncture under anesthesia. Serum AST, ALT and total bilirubin were determined by the Clinical Veterinary Haematology Service (Universitat Autónoma de Barcelona, Spain).

### Statistical analysis

Results are expressed as mean ± SEM of at least three independent experiments. Statistical differences were determined using Prism (version 5; GraphPad software). Unless otherwise stated differences between experimental groups were analyzed by the non-parametric Mann-Whitney *U* test. Differences between *in vivo* tumor growth curves were analyzed by a linear regression model. The level of significance was considered for *P* values less than 0.05.

## SUPPLEMENTARY MATERIALS FIGURES AND TABLES


