# Supplementary material for: A NOTCH-sensitive uPAR-regulated oncolytic adenovirus effectively suppresses pancreatic tumor growth and triggers synergistic anticancer effects with gemcitabine and nab-paclitaxel
Source: Oncotarget. 2017 Feb 7;8(14):22700–15. doi: 10.18632/oncotarget.15169 (PMC5410256; doi:10.18632/oncotarget.15169)
Supplement: Supplementary file 1 [file oncotarget-08-22700-s001.pdf]

A Notch-sensitive uPAR-regulated oncolytic adenovirus effectively suppresses pancreatic tumor growth and triggers synergistic anticancer effects with gemcitabine and nab-paclitaxel

Supplementary Materials

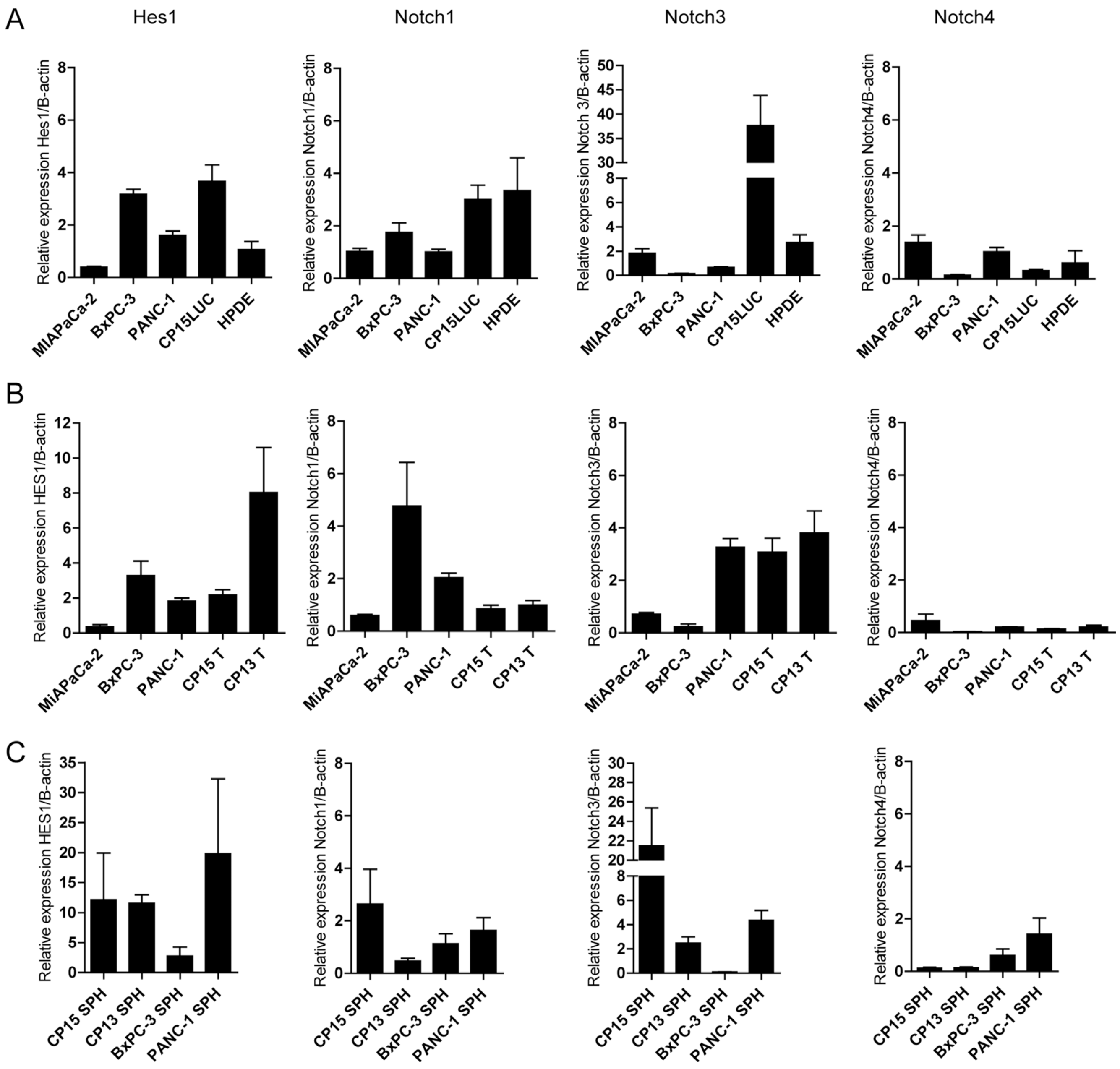

Supplementary Figure 1: Expression levels of Notch related genes in pancreatic cancer models. mRNA expression levels of Hes1, Notch1, Notch3 and Notch4 in pancreatic cancer cell lines (**A**), in subcutaneous tumor models (**B**) and in tumorsphere cultures (**C**). mRNA expression levels are normalized to β-actin expression. Results are expressed as the mean +/– SEM of at least three independent experiments.

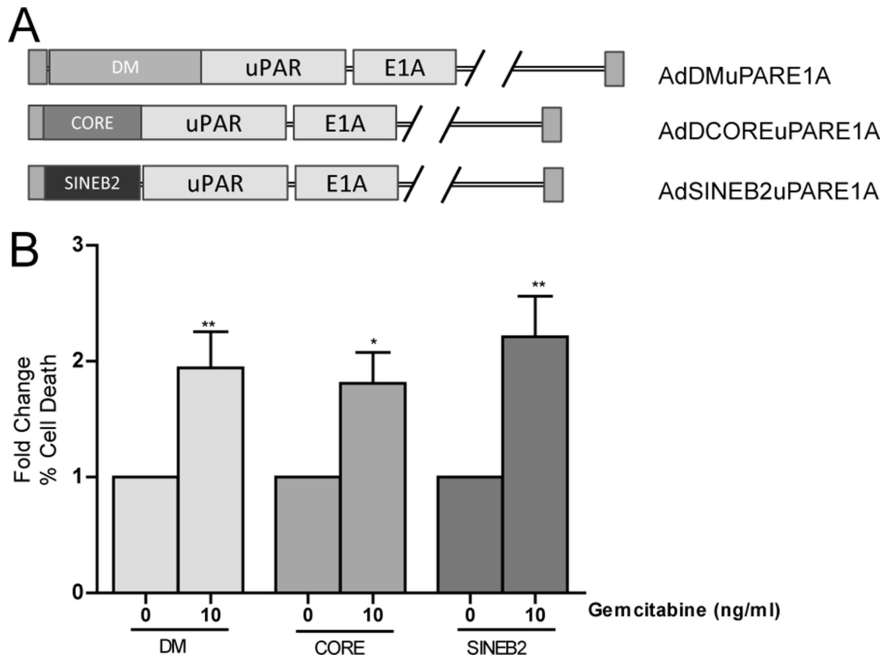

Supplementary Figure 2: (**A**) Schematic representation of AduPARE1A-derived oncolytic adenoviruses containing insulator sequences. (**B**) Percentage of cell death in BxPC-3 cultures infected with 10 MOI of the indicated adenoviruses and treated, or not, with 10 ng/mL of gemcitabine for 24 h. Cell death was analyzed 72 h after infection by MTT assay and results were relative to mock treated cells. Bar graph shows the fold increase in cell death in the presence of gemcitabine. Results are represented as the mean +/– SEM of four independent experiments

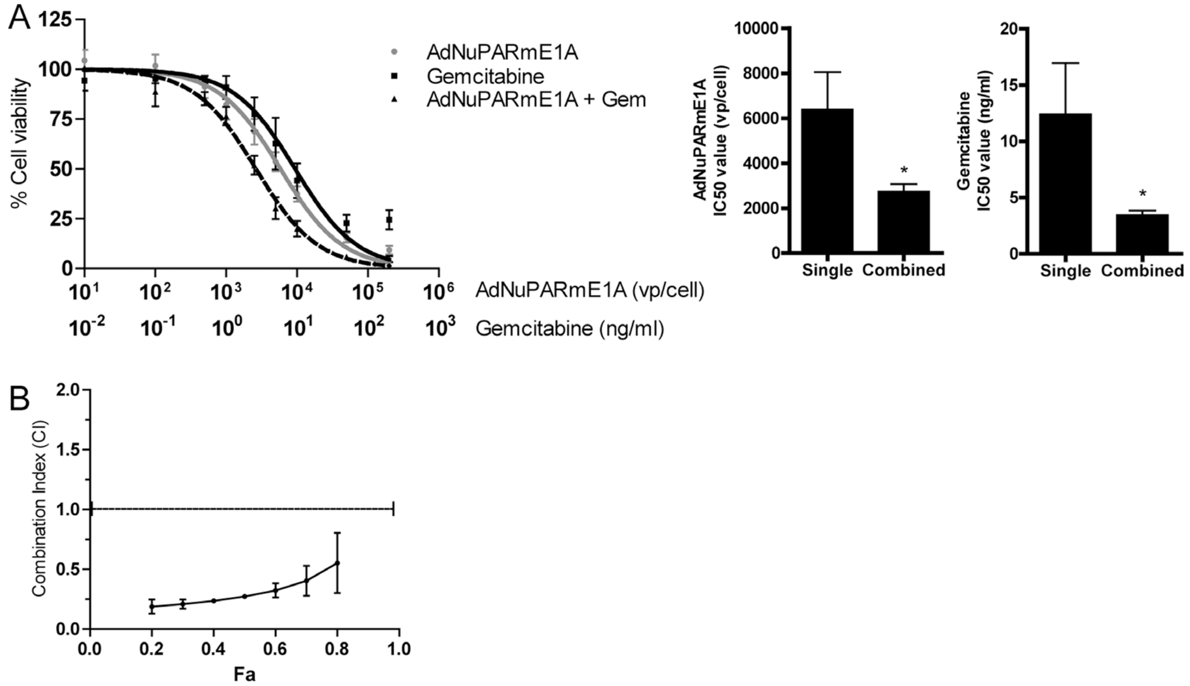

Supplementary Figure 3: (**A**) Dose –response curves of gemcitabine, AdNuPARmE1A or combined treatment in BxPC-3. Cells were seeded in triplicate and treated with a dose range of gemcitabine (ng/mL) and/or AdNuPARmE1A (vp/cell). Cell viability was measured 72 h later by MTT assay and is expressed as cell viability normalized to mock treated cultures. IC50 values for monotherapy or combination therapy for each treatment are represented in bar graphs. (**B**) Combination index values (CI) for the interaction of gemcitabine and AdNuPARmE1A are calculated as a function of inhibitory fractions. Results are expressed as a mean +/– SEM of four independent experiments.

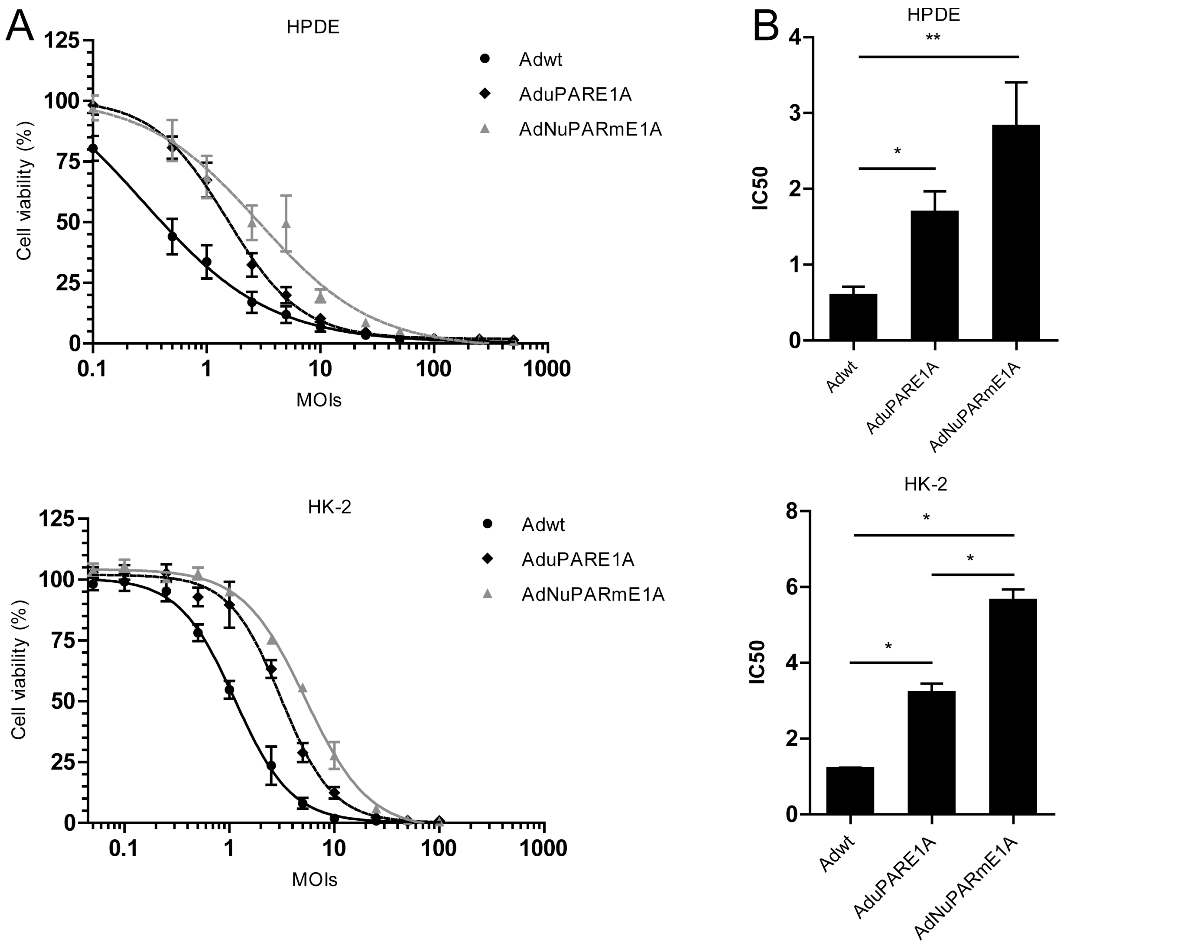

Supplementary Figure 4: Cytotoxicity of Adwt, AduPARE1A and AdNuPARmE1A in HPDE and HK-2 non-tumoral models at 7 days post-infection. Cells were seeded in triplicates and treated with a dose range of adenoviruses (pfu/cell). Cell viability was measured 7 days post-infection and normalized to mock treated cultures. (**A**) Dose-response curves. (**B**) IC50 values. Results are expressed as a mean +/– SEM, of at least 6 independent experiments.

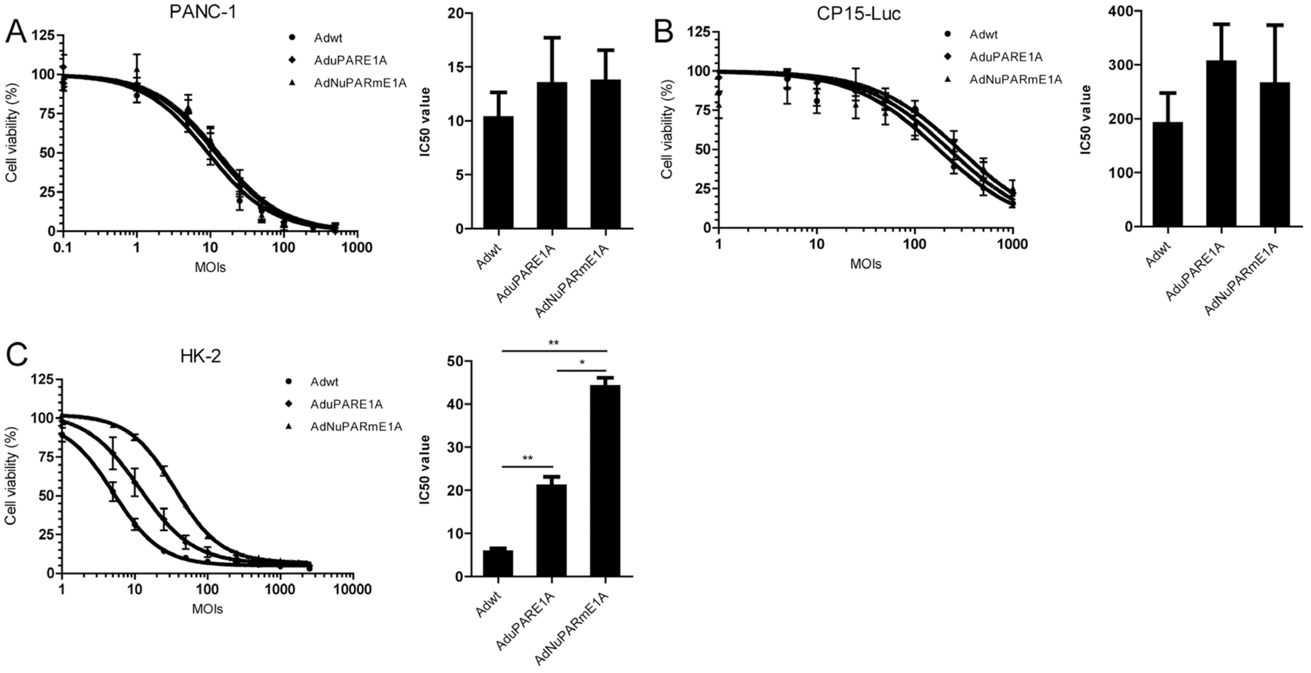

Supplementary Figure 5: Cytotoxicity of Adwt, AduPARE1A and AdNuPARmE1A in pancreatic cancer and non-tumoral cell models. Cells were seeded in triplicate and treated with a dose range of adenoviruses (pfu/cell). Cell viability was measured 72 h post infection by MTT assay and normalized to mock treated cultures. (**A**) PANC-1, (**B**) CP15-Luc, (**C**) HK-2. Left pannel represents dose-response curves. Right panel shows IC50 values. Results are expressed as a mean +/– SEM, of at least 4 independent experiments.

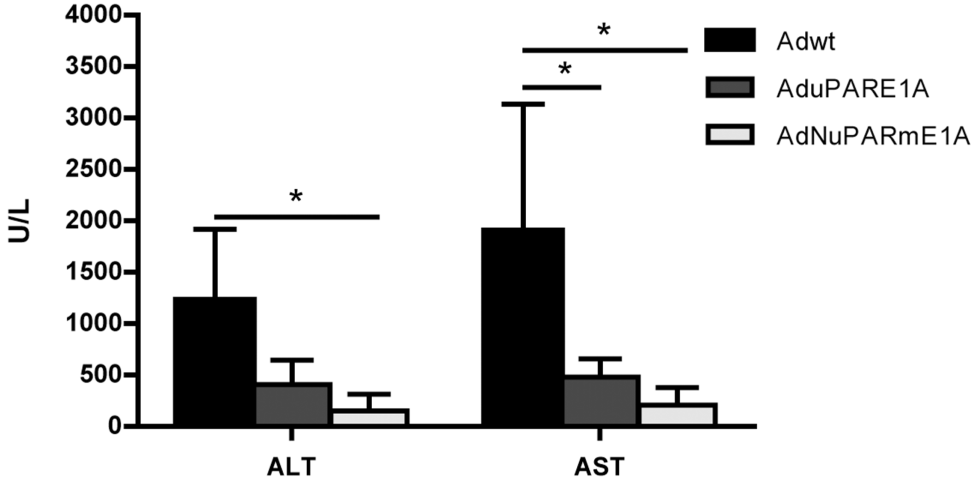

Supplementary Figure 6: Hepatotoxicity of Adwt, AduPARE1A, and AdNuPARmE1A after systemic administration in immunocompetent mice. Mice (n = 5/group) received (2×10^{10} vp/mouse) of the indicated virus. Serum transaminases ALT and AST were measured three days later.

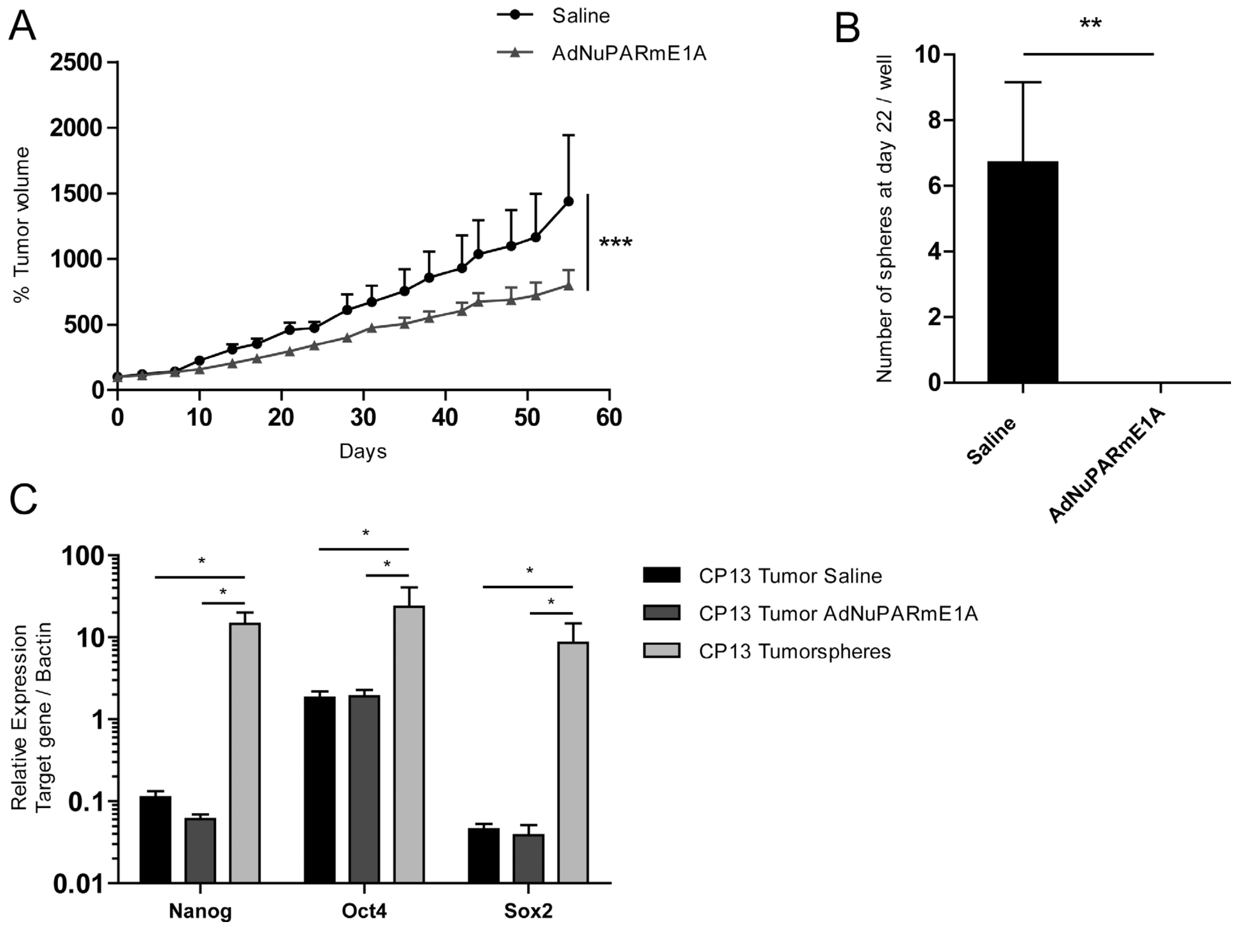

Supplementary Figure 7: *In vivo* antitumoral activity of AdNuPARmE1A in CP13 PDX tumors and cancer stem cell analysis. (**A**) Mice bearing subcutaneous tumor fragments of CP13 PDX tumors were treated with a single dose of AdNuPARmE1A (5×10^{10} vp) or with saline in the control group. A. Follow up of CP13 PDX tumor volume (mm3) as growth percentage, ($n \geq 9$). (**B**) Tumorspheres grown as anchorage-independent colonies from CP13 saline or adenovirus-treated tumors. Quantification of the number of tumorspheres at day 22 after seeding. Results are expressed as the mean +/– SEM of $n = 7$ tumors/treatment (**$p < 0.01$). (**C**) RT-qPCR analysis of pluripotency-associated genes from tumors and tumorspheres at day 22. Results are expressed as the mean +/– SEM of $n \geq 3$ (*$p < 0.05$).

Supplementary Table 1: Primer list

GENES	PRIMER SEQUENCES	AMPLICON SIZE
Hexon	Fw 5′- GCCGCAGTGGTCTTACATGCACATC -3′	301 bp
	Rv 5′- CAGCACGCCGCGGATGTCAAAG -3′	
E1A	Fw 5′- ATCGAAGAGGTACTGGCTGA -3′	405 bp
	Rv 5′- CCTCCGGTGATAATGACAAG-3′	
Notch1	Fw 5′- ATCCAGAGGCAAACGGAG-3′	106 bp
	Rv 5′- CACATGGCAACATCTAACCC-3′	
Notch3	Fw 5′- AGATTCTCATCCGAAACCGCTCTA-3′	259 bp
	Rv 5′-GGGGTCTCCTCCTTGCTATCCTG -3′	
Notch4	Fw 5′- TGCGAGGAAGATACGGAGTG-3′	116 bp
	Rv 5′- CGGGATCGGAATGTTGG-3′	
Hes1	Fw 5′-TGGAAATGACAGTGAAGCACCT -3′	116 bp
	Rv 5′-GTTCATGCACTCGCTGAAGC -3′	
Nanog	Fw 5′-TGAACCTCAGCTACAAACAGGT-3′	312 bp
	Rw 5′-AACTGCATGCAGGACTGCAGAG-3′	
Oct4	Fw 5′-CTTGCTGCAGAAGTGGGTGGAGGAA-3′	123 bp
	Rw 5′-CTGCAGTGTGGGTTTCGGGCA-3′	
Sox2	Fw 5′-AGAACCCCAAGATGCACAAC-3′	114 bp
	Rw 5′-CGGGGCCGGTATTTATAATC-3′	
GDX	Fw 5′-GGCAGCTGATCTCCAAAGTCCTGG-3′	240 bp
	Rv 5′-AACGTTCGATGTCATCCAGTGTTA-3′	
B-actin	Fw 5′- CTGGAACGGTGAAGGTGACA-3′	195 bp
	Rv 5′- GGGAGAGGACTGGGCCATT-3	
